# Antioxidant, antimicrobial and neuroprotective effects of Octaviania asterosperma in vitro

**DOI:** 10.1080/21501203.2020.1816584

**Published:** 2020-12-02

**Authors:** Mustafa Sevindik, Hasan Akgul, Zeliha Selamoglu, Nady Braidy

**Affiliations:** a Bahçe Vocational High School, Osmaniye Korkut Ata University, 80500, Osmaniye, Turkey; b Department of Biology, Faculty of Science, Akdeniz University, Antalya, Turkey; c Department of Medical Biology, Faculty of Medicine, Nigde Ömer Halisdemir University, Nigde, Turkey; d Centre for Healthy Brain Ageing, School of Psychiatry, University of New South Wales, Sydney, Australia

**Keywords:** *Octaviania asterosperma*, phenolic compounds, antioxidant, antimicrobial, oxidant, L-glutamate

## Abstract

**Octaviania asterosperma**: (hypogeous Basidiomycota) We investigated the phenolic composition, and antioxidant, antimicrobial and antigenotoxic effects of methanol extracts of fruiting bodies from Octaviania asterosperma. The total phenolic content (ppm) of O. asterosperma was found to be catechin (54.73 ± 4.68), epicatechin (123.90 ± 8.52), caffeic acid (4.23 ± 0.97), p-hydroxybenzoic acid (37.72 ± 3.84), cinnamic acid (58.07 ± 5.40), gallic acid (56.64 ± 6.39), clorogenic acid (80.76 ± 4.92) and coumaric acid (2.45 ± 0.15). The total antioxidant status (TAS), total oxidant status (TOS) and oxidative stress index (OSI) were 3.410 ± 0.099 mmol/L, 7.548 ± 0.147 μmol/L and 0.221 ± 0.005 respectively. O. asterosperma showed some promising antimicrobial activity. The extract showed no genotoxic potential and attenuated hydrogen peroxide (H2O2)-induced oxidative DNA damage in neurons. Pre-treatment with O. asterosperma maintained mitochondrial function, reduced expression levels of cleaved-caspase-3 and apoptosis-inducing factor (AIF) when HT22 cells were exposed to pathophysiological concentrations of GLU (25 mM) and modulated protein kinase B (Akt), the mammalian target of rapamycin (mTOR), and the phosphotase and tensin homolog on chromosome ten (PTEN). O. asterosperma is an important food for the treatment or management of neurodegenerative disorders due to its phenolic content and potent antioxidant and anti-excitotoxic effects.

## 1. Introduction

Alzheimer’s disease (AD) is a complex neurodegenerative disorder that is associated with ageing as a major risk factor. AD is characterized by the progressive loss of cholinergic neurons leading to cognitive decline and mortality (Fish et al. 2019). Although the exact pathobiology of disease remains unclear, neuronal loss due to oxidative and excitotoxic mechanisms has been hypothesized to play a major role (Martini et al. 2019). In AD, markers for oxidative stress and mitochondrial dysfunction have been reported in close proximity to key pathological hallmarks of AD, including extracellular amyloid-beta (Aβ) deposits, and intracellular neurofibrillary tangles containing hyperphosphorylated tau protein (Birnbaum et al. 2018; Butterfield and Boyd-Kimball 2018; Cheignon et al. 2018; Martins et al. 2018). Mitochondrial dysfunction represents an important feature in AD pathology and can enhance further production of highly volatile reactive oxygen species (ROS), which can further stimulate accumulation of Aβ and tau hyperphosphorylation (Canevari et al. 2004; Ansari et al. 2006; Todd et al. 2016).

Moreover, the main excitatory neurotransmitter in the central nervous system (CNS), glutamate (GLU), has been shown to maintain neuronal and synaptic function. However, pathophysiological concentrations of GLU have been shown to inhibit endogenous antioxidant defense mechanisms and enhance ROS production (Babu and Bawari 1997; Duarte et al. 2000; Salustri et al. 2010; Shah et al. 2016; Rosa et al. 2018). GLU-induced toxicity in the mouse hippocampal neuronal cell line, HT22, represents an important model to investigate the neuroprotective effects of naturally-occurring and synthetic agents in neurodegenerative diseases (Lee et al. 2010; Sucontphunt et al. 2011; Yue et al. 2016). We have previously demonstrated that naturally occurring green tea polyphenols (i.e. epigallocatechin gallate (EPCG), catechin hydrate, curcumin, apigenin, naringenin and gallotannin) can inhibit specific excitotoxic processes such as calcium (Ca^2+^) influx, nitric oxide synthase (NOS) activity, and oxidative DNA damage (Braidy et al. 2010). Our work and others provides rationale for the beneficial health effects of polyphenols in excitable tissue, and the brain in particularly.

At present, there are no effective treatments available for AD patients in the clinic (Fish et al. 2019). However, numerous epidemiological studies and clinical trials has shown that supplementation with herbs and fungi rich in polyphenols and antioxidants may have beneficial effects in age-related diseases and exert favourable safety profiles (Rossi et al. 2008; Luo et al. 2009; Thomas et al. 2009; Fernandez-Fernandez et al. 2012; Pasinetti 2012; Malar and Devi 2014; Lakey-Beitia et al. 2015; Pasinetti et al. 2015; Dal-Pan et al. 2017; Jabir et al. 2018; Silveira et al. 2018). It is well established that some mushrooms are effective ROS scavengers, redox-active metal chelators, and potent inhibitors of lipid peroxidation and protein carbonyl formation, and can increase the levels of endogenous antioxidants and activity of antioxidant enzymes (Liu et al. 2004; Asatiani et al. 2007; Oyetayo 2009). In addition to these beneficial effects, a polysaccharide isolated from *Amanita caesarea* has been shown to protect against GLU toxicity in PC12 cells by improving mitochondrial function and attenuating apoptosis (Dogan and Akbas 2013). Moreover, extracts from *Hericum erinaceus* have been shown to protect against AD not only through regulation of mitochondria-mediated apoptosis, but also via modulation of major neurotransmitters (Zhu et al. 2016). However, the potential neuroprotective effects of *Octaviania asterosperma* have not to our knowledge been previously investigated.

The genus *Octaviania* is widespread in Europe, although it has been placed on the red lists of threatened species in some countries that are mostly edible. Ther are found as ectomycorrhizal to broad-leaved trees (Venturella et al. 2011). However, information regarding the constituents and biological activity of these mushrooms is nascent in the current literature. In the present study, we aimed to determine the phenolic composition of *O. asterosperma* mushrooms collected from northwest Turkey. We also assessed its antioxidant, antimicrobial and antigenotoxic capacity *in vitro*. We also examined the neuroprotective effects of these mushrooms against GLU-induced apoptosis in HT22 murine hippocampal neuronal cells. Our current study suggests that *O. asterosperma* mushrooms may be serve important roles as functional foods for a variety of acute and chronic conditions including AD.

## 2. Materials and methods

### 2.1 Mushroom species

The specimen was collected from Bursa province in Turkey. Morphological and ecological characteristics of the samples were noted and photographed in the field. Measurements of microscopical features were taken on dry materials mounted in KOH and Melzer’s reagent and confirmation of the mushrooms were made by mycological experts at Akdeniz University, Turkey. The dried samples were conserved in fungarium of Akdeniz University. The names of taxa and authors are quoted according to MycoBank (www.mycobank.org) and Index Fungorum (www.indexfungorum.org).

### 2.2 Extraction

Mushroom specimens were dried in the laboratory under suitable conditions. The powder samples were weighed and 30 g was extracted with 250 mL methanol (MeOH) and 250 mL dichloromethane (DCM) in the soxhlet apparatus. (Gerhardt EV 14). The obtained extracts were concentrated using a rotary evaporator (Heidolph Laboratory 4000 Rotary Evaporator).

### 2.3. Determination of TAS, TOS and OSI

The mushroom total antioxidant status (TAS), total oxidant status (TOS) levels and oxidative stress index (OSI) were determined using the Rel Assay kit (Rel Assay Kit Diagnostics, Turkey). Trolox was used as the calibrator in the TAS analysis, and hydrogen peroxide was used as the calibrator in the TOS studies (Erel 2004, 2005). To determine the OSI, the mmol unit of TAS and μmol unit of the TOS were cross-converted and the index value was expressed as percentage (Erel 2005). TAS and TOS tests were conducted on 5 mushroom samples in 5 replicates.

### 2.4. Antimicrobial activity tests

Antimicrobial activity tests were conducted with the agar dilution method recommended by the Clinical and Laboratory Standards Institute (CLSI) and the European Committee on Antimicrobial Susceptibility Testing (EUCAST) on mushroom MeOH and DCM extracts. Minimal inhibitor concentration (MIC) for each extract was determined against standard bacterial and fungal strains. *Staphylococcus aureus* ATCC 29,213, *Staphylococcus aureus* MRSA ATCC 43,300, *Enterococcus faecalis* ATCC 29,212 were used as gram positive bacteria. *Escherichia coli* ATCC 25,922, *Pseudomonas aeruginosa* ATCC 27,853 and *Acinetobacter baumannii* ATCC 19,606 were used as gram negative bacteria. *Candida albicans* ATCC 10,231, *Candida krusei* ATCC 34,135 ATCC 13,803 and *Candida glabrata* ATCC 90,030 were used as fungi. Bacterial strains were pre-cultured in Muller Hinton Broth medium and fungal strains were pre-cultured in RPMI 1640 Broth medium. To obtain standard inoculum, the turbidity of the bacteria and fungi was designed based on the McFarland 0.5 scale. All extracts were tested at concentrations of 800–12.5 μg/mL and all dilutions were prepared with distilled water. Solvents used for the extraction were also tested for antimicrobial activity. Fluconazole, amphotericin B were used as reference drugs for the fungi and amikacin, ampicillin and ciprofloxacin were used as reference drugs for the bacteria. The minimal dilution that inhibited the growth of bacteria and fungi was identified as the minimum inhibitory concentration (MIC). (Bauer et al. 1966; Hindler et al. 1992; Matuschek et al. 2014).

### 2.5. Determination of phenolic compounds

Mushroom extracts were processed using a modified version of the method developed by Caponio et al. (1999) using a SHIMADZU system HPLC device and a DAD detector (Caponio et al. 1999): Injection volume was adjusted to 20 μL. 3% acetic acid was used as mobile phase A and methanol was used as mobile phase B and the flow rate was reg-ulated to 0.8 mL per minute. Chromatographic separation was conducted with Agilent Eclipse XDB-C18 column (250x4.6 mm id 5 μM) at 30°C.

### 2.6. Cell culture

HT22 mouse hippocampal neuronal cells (BNCC, 337,709) were cultured in a cell culture flask at a density of 1 × 10^5^ cells/mL and grown in Dulbecco’s Modified Eagle Medium (DMEM) supplemented with 10% fetal calf serum (FCS), 1% L-glutamax, and 1% antibiotic/antifungal in an atmosphere containing 5% CO_2_ and 95% oxygen. All cell culture equipment were obtained from Invitrogen (Melbourne, Australia).

### 2.7. Cell viability assay

MTT (3- [4, 5-dimethylthi-azol-2-yl]- 2, 5-diphenyl-tetrazolium bromide) was used to measure cell viability in HT22 cells (Caponio et al. 1999). In short, cells were detached using 3.0 mL Trypsin-EDTA so-lution (Sigma-Aldrich, MO, USA) after reaching 70–80% confluency and cultured in 24-well plates and incubated for 24 hours. After 24 hours, varying dilutions of (25, 50, 100, 200 μg/ml) the extracts were applied, and cells were incubated for a further 24 hours. Control cells were only treated with the growth medium. After 48 hours of incubation, the supernatants were replaced with 1 mg/mL MTT (Sigma) dis-solved in growth medium and incubated at 37°C until purple precipitate was visible. Following incubation, supernatants were removed and MTT absorbed by cells was dissolved by adding dimethyl sulfoxide (DMSO) (Sigma-Aldrich, MO, USA). Plates were read at 570 nm using an Epoch spectrophotometer (BioTek Instruments, Winooska, VT).

### 2.8. Antigenotoxicity assay

The antigenotoxic effects of mushroom MeOH and DCM extracts on DNA damage were determined using an in vitro DNA cleavage assay using pBR 322 plasmid DNA (Mishra et al. 2011). Briefly, 25, 50, 100 and 200 μg/mL standard solutions were prepared with mushroom extracts. 0.5 μg plasmid pBR 322 was added to super-coil DNA Eppendorf tubes and 10 μL standard mushroom extract solution was added. 10 μL of Fenton’s agent (30 mM H_2_O_2_, 50 μM ascorbic acid and 80 μM FeCl3) was added to the prepared solution and the product was incubated for 10 minutes at ambient temperature. The final volume of the mixture was adjusted to 20 mL and allowed to stand for 30 minutes at 37°C. The DNA was then analysed by electrophoresis on a 1% agarose gel containing ethidium bromide.

### 2.9. Mitochondrial function assay

To determine the effect of mushroom extracts on oxygen consumption rates (OCRs; as indicator of mitochondrial respiration) in HT22 cells, the Seahorse XF24, extracellular flux analyzer (Seahorse Bioscience, North Billerica, MA, USA) was employed as previously described (Schuh et al. 2011). After determination of the basal respiration in the cell culture, oligomycin (2 µM), carbonylcyanide-p-trifluoromethoxy-phenylhydrazone (FCCP, 500 nM), and antimycin (3 µM) were added and the oxygen consumption rates (OCRs) for each culture well were quantified for 2 minutes. This allowed us to determine the basal control ratio (BCR i.e. Basal/maximum respiration) and the uncoupling ratio (UCR i.e. mitochondrial functional integrity) (Pesta and Gnaiger 2012). Essentially, the BCR is a measurement of how close the basal level of respiration is to the maximum level of respiration (i.e., basal/maximum).

### 2.10. Western blotting to determine protein expression of cleaved caspase-3, AIF, calpain-1, phosphorylated P-PTEN, total T-PTEN, phosphorylated P-AKT, total T-AKT, phosphorylated P-mTOR, total T-mTOR

HT22 cells were pretreated with on ice and immediately processed. Briefly, cells were harvested and homogenized in RIPA buffer (50 mM, Tris-Cl, pH 7.5, 150 mM NaCl, 1% NP-40, 0,5% sodium deoxycholate, and 1% SDS), supplemented with a protease inhibitor cocktail (Sigma-Aldrich P8340) and phosphatase inhibitors (50 mM NaF, 1 mM Na_3_VO_4_ and 30 μM Na_4_P_2_0_7_). Protein samples were centrifuged at 14,000 rpm at 4°C twice for 15 min [26]. Total protein concentration was determined using the BCA Protein Assay Kit (Pierce Biotechnology, Rockford, IL). Protein samples (20 µg) were resolved by 10% SDS-PAGE and transferred to a PVDF membrane. The incubation with a primary antibody at 4°C cleaved caspase 3 (ab2302), calpain-1 (bs-1099 R), AIF (bs-0037 R), glyceraldehyde-3-phosphate dehydrogenase (GAPDH; ab109268), p-PTEN (bs-3350 R), t-PTEN (bs-0686 R), p-Akt (S473), t-Akt (ab106693), p-mTOR (ab83495), and p-mTOR (S2448). Aftewards, a secondary anti-goat peroxidase conjugated antibody (Pierce) was used and developed using an ECL kit (Western Lightning Plus ECL, PerkinElmer) following the manufacturer’s instructions.

### 2.11. Statistical analysis

All experiments were performed 5 times and results expressed as mean ± standard deviation unless otherwise stated. Statistical analysis using Student’s *t*-test was performed using Microsoft Excel. Results were considered significant when *p* < 0.05.

## 3. Results and discussion

### 3.1. Phenolic content of O. asterosperma

**Figure 1.**
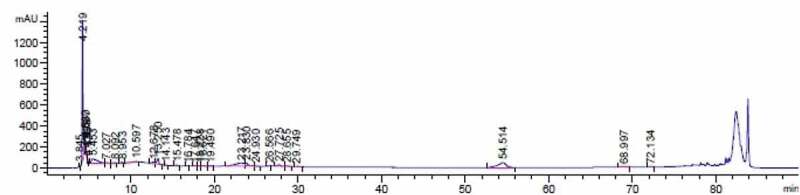
Chromatogram obtained by the separation of *Octaviania asterosperma* methanol extract

In this study, the phenolic content of *O. asterosperma* mushroom methanol extracts were determined using HPLC and the findings are presented in Table 1 and Figure 1. Analysis of phenolic compounds demonstrated that there are at least 8 main polyphenolic substances, notably, was found to be catechin (54.73 ± 4.68), epicatechin (123.90 ± 8.52), caffeic acid (4.23 ± 0.97), p-hydroxybenxoic acid (37.72 ± 3.84), cinnamic acid (58.07 ± 5.40), gallic acid (56.64 ± 6.39), chlorogenic acid (80.76 ± 4.92) and coumaric acid (2.45 ± 0.15). It is well-established that catechin and its derivative epicatechin are potent antioxidant compounds that are involved in the inhibition of free radicals. They have also been shown to improve AD-like pathology in various in vitro and in vivo models through inhibit mitochondrial dysfunction, reduce oxidative stress and inflammation, maintain intracellular Ca^2+^ signaling, and modulate autophagic pathways (Ejaz Ahmed et al. 2013; Lim et al. 2013; Suganthy et al. 2016). Chlorogenic and caffeic acid have been shown to cross the blood-brain barrier, and exert neuroprotective effects in brain tissue (Oboh et al. 2013). As well, the antiamyloidogenic and antiapoptotic effects of chlorogenic acid in brain cell cultures has been well demonstrated (Yang et al. 2018). Chlorogenic acid has been to improve brain function by reducing the activities of acetylcholinesterase and butyrylcholinesterase in rat brain homogenates. These enzymatic activities are increased in AD, and inhibition of these enzymatic activities can increased synaptic levels of the neurotransmitter acetylcholine (Agunloye et al. 2019). Hydroxybenzoic acid and gallic acid have been demonstrated to be effective scavengers of free radicals and reactive nitrogen species, including peroxynitrite (Shah and Verma 2011, 2012). Peroxynitrite, which is produced from the reaction of nitric oxide and the highly volatile superoxide, can induce lipid peroxidation, and other deleterious processes leading to inactivation of major metabolic enzymes, release of proapoptotic factors, and activation of stress signalling pathways associated with AD (Smith et al. 1997; Van Dyke 1997; Paris et al. 1998; Koppal et al. 1999; Reynolds et al. 2005). Apart from its potent antioxidant and anti-inflammatory effects, cinnamic acid has been shown to activate the nuclear hormone receptor PPAR-alpha to transcriptionally upregulate the expression of a master regulator Transcription factor EB (TFEB) to stimulate lysosomal biogenesis (Chandra et al. 2018). This has important therapeutic implications for the treatment of AD and other neurodegenerative diseases associated with the accumulation of toxic protein aggregates and impaired lysosomal function. Coumaric acid has been recently shown to inhibit the expression of iNOS and the pro-inflammatory enzyme COX-2 in PC12 cells exposed to the Aβ peptide, and inhibited NF-KB activity (Yoon et al. 2014). Therefore adequate consumption of *O. asterosperma* mushroom is important due to its rich phenolic content and their beneficial effects in AD.

**Table 1.** Phenolic contents of mushroom.

	*O. asterosperma* (ppm)
Gallic acid	56.64
Catechin	54.73
Epicatechin	123.90
Chlorogenic acid	80.76
Coumaric acid	2.45
Caffeic acid	4.23
p-Hydroxybenzoic acid	37.72
Cinnamic acid	58.07

### 3.2. TAS, TOS and OSI values for O. asterosperma

In the present study, the TAS (mmol/L), TOS (μmol/L) and OSI values we also evaluated using the *O. asterosperma* ethanol extract. The findings are presented in Table 2. As shown in Table 2, the TAS value of the mushroom ethanol extract was 3.410 ± 0.099 mmol/L. The TAS value reported in this study is much higher than the TAS value reported in other mushroom extracts: *Fomitopsis pinicola, Laetiporus sulphureus, Infundibulicybe geotropa* and *Cerioporus varius* was 1.44, 2.195, 1.854 and 2.312 mmol/L respectively (Sevindik et al. 2017, 2018, 2020; Sevindik 2019). This may be due to the greater phenolic content of the mushroom extract under investigation. This results, it also shows that it produces more antioxidant compounds in *O. asterosperma* than other mushroom.

**Table 2.** TAS, TOS and OSI values of mushroom.

	TAS (mmol/L)	TOS(μmol/L)	OSI
*O. asterosperma*	3.410 ± 0.099	7.548 ± 0.147	0.221 ± 0.005

The TOS value of *O. asterosperma* ethanol extract was 7.548 ± 0.147 μmol/L. This was much lower compared to the TOS value reported for *F. pinicola, I. geotropa* and *C. varius* (14.21, 30.38 and14.36, respectively), but higher than *L. sulphureus*, (1.303 μmol/L) (Sevindik et al. 2017, 2018, 2020; Sevindik 2019). Differences between mushroom TOS values may be attributed to the different methodologies used by different research groups at different times and the different species of mushroom collected in different locations. It has been suggested that mushrooms with high TOS values should be cautiously consumed, since they are capable of producing free radicals to protect against harmful endogenous factors present in the environment. Therefore, our findings suggest that *O. asterosperma* mushrooms may be the preferred mushroom to consume due to its higher TAS and lower TOS value compared to other mushrooms present in Turkey and other geographical regions.

It is well established that the OSI value reflects the rate of inhibition of the oxidant compounds by mushrooms due to environmental and/or inherent factors by the antioxidant compounds present in the organism. The OSI value for *O. asterosperma* mushroom reported in this study was 0.221 ± 0.005. On the other hand, the OSI was 0.99, 0.059, 1.639 and 0.627 for *F. pinicola, L. sulphureus, I. geotropa* and *C. varius* respectively (Sevindik et al. 2017, 2018, 2020; Sevindik 2019). The OSI for *O. asterosperma* was lower than that of *F. pinicola, I. geotropa and C. varius* mushrooms and higher than *L. sulphureus*. Oxidative stress induced by oxidant molecules was prevented by TAS, which covers the whole enzymatic and non-enzymatic systems, thus leading to lower OSI values.

### 3.3. Antimicrobial activity of O. asterosperma

We also examined the antimicrobial activity of MeOH and DCM *O. asterosperma* extracts were determined (Table 3). As shown in Table 3, DCM extracts of *O. asterosperma* extracts were more effective than MeOH for most tested microorganisms. It was found that these extracts were active when compared to other extracts. *I. geotropa* extracts generally appeared to be more effective against all tested strains including *E. faecalis, P. aeruginosa, A. baumannii, C. albicans, C. krusei* and *C. glabrata*. Taken together, *O. asterosperma* has some antimicrobial potential which may be clinically relevant.

**Table 3.** Antimicrobial activities of mushroom.

	*S. aureus*	*S. aureus* MRSA	*E. faecalis*	*E. coli*	*P. aeruginosa*	*A. baumannii*	*C. albicans*	*C. glabrata*	*C. krusei*
MeOH	25	25	50	25	50	50	25	50	25
DCM	50	50	100	50	50	100	50	50	50
Ampicillin	1.56	3.12	1.56	3.12	3.12	-	-	-	-
Amikacin	-	-	-	1.56	3.12	3.12	-	-	-
Ciprofloksasin	1.56	3.12	1.56	1.56	3.12	3.12	-	-	-
Flukanazol	-	-	-	-	-	-	3.12	3.12	-
Amfoterisin B	-	-	-	-	-	-	3.12	3.12	3.12

### 3.4. Antigenotoxicity of O. asterosperma

**Figure 2.**
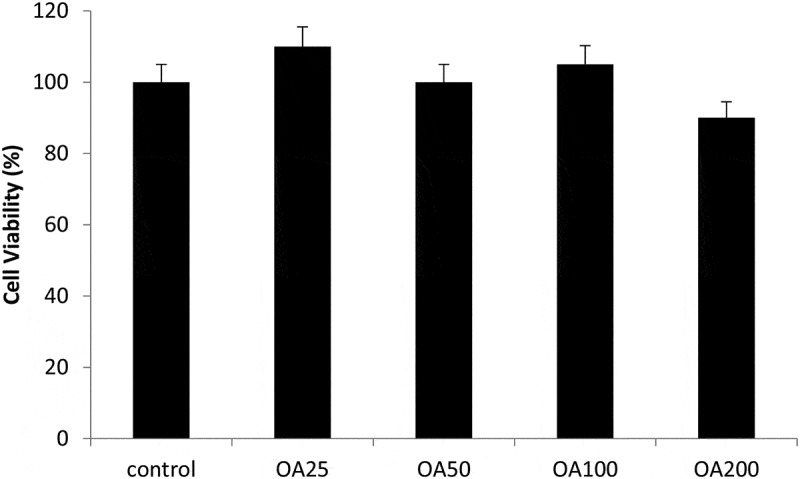
Viability of HT22 cells after 24 h treatment with *Octaviania asterosperma extract* (25, 50, 100 and 200 µg/ml)

**Figure 3.**
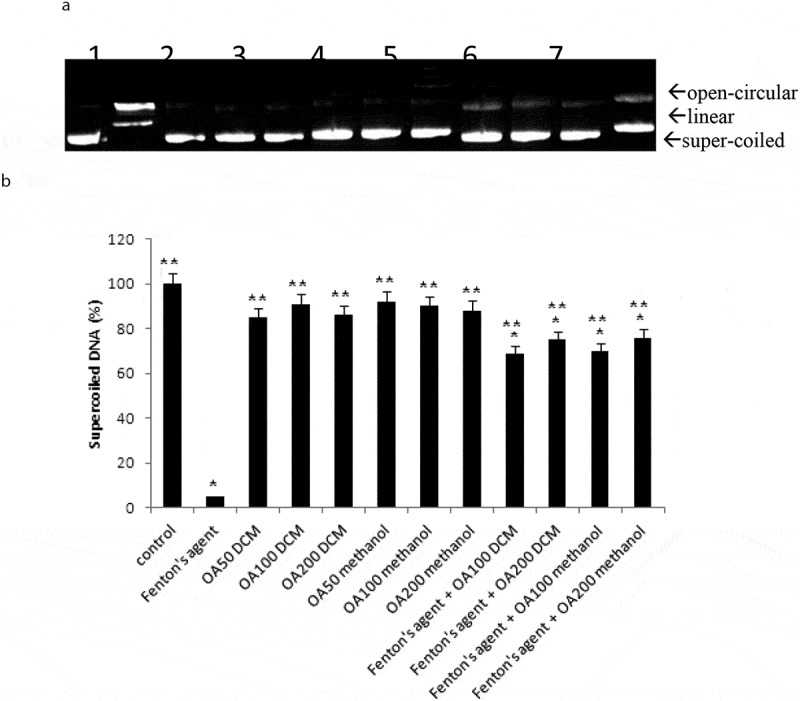
Antigenotoxic effects of *Octaviania asterosperma extract* (50, 100 and 200 µg/ml)

Our study also investigated the effects of *O. asterosperma* in HT22 cells. Our data shows that the MeOH *O. asterosperma* extract displayed no toxicity at 25–200 µg/mL after 24 h (Figure 2). On the contrary, some toxicity has been previously reported by other mushroom extracts eg *A. blazei, G. frondosa* and *H. erinaceus* in Chinese hamster fibroblast cells after 24 h at concentrations of 2 mg/mL (Aprotosoaie et al. 2017) (Figure 3). We also investigated the ability of *O. asterosperma* to induce DNA damage using the pBR322 plasmid DNA. The conversion of the supercoiled form of DNA of the plasmid to an open-circular or linear form of DNA has been used as an index of DNA damage (Mishra et al. 2011). Our data shows that in the absence of H_2_O_2_ or any treatment, the pBR322 plasmid DNA was in the supercoiled form. However, treatment with H_2_O_2_ led to a reduction in the supercoiled DNA and increase formation of relaxing open-circular and linear forms. At the concentrations of 50–200 µg/ml, no significant shift in the DNA fragments from the supercoiled form to either open-circular and linear forms suggesting that *O. asterosperma* extracts have little effect on genotoxicity. Treatment with *O. asterosperma* extracts and H_2_O_2_ led to a reduction in the formation of relaxing open-circular and linear forms of DNA suggesting that the potential protective effect against oxidative DNA damage by this extract. While most edible mushrooms to do not exhibit genotoxic potential, some extracts e.g. *A. blazei* have been shown to be genotoxic to HTC rat hepatoma cells (Bellini et al. 2006). The genotoxic potential of mushrooms is necessary to establish a safety profile of mushrooms for human consumption. The limited toxicity and genotoxic effects of *O. asterosperma* extracts in murine HT22 neuronal cells is favourable and warrants further investigation.

Results are presented as mean ± SE of 5 independent experiments. *p < 0.05 when compared with control (t-test)

(a) Image of agarose gel of pBR322 DNA showing bands of open-circular, linear and supercoiled DNA. Lanes on gel represent: (Lane 1) control DNA (no treatment); (Lane 2) Fenton’s agent; (Lane 3–5) 50, 100 and 200 µg/ml *Octaviania asterosperma* DCM extract, (Lanes 6–8) 50, 100 and 200 µg/ml *Octaviania asterosperma* methanol extract, (Lanes 9–10) Fenton’s agent and 100 and 200 µg/ml *Octaviania asterosperma* DCM extract respectively, (Lanes 11–12) Fenton’s agent and 100 and 200 µg/ml *Octaviania asterosperma* methanol extract respectively, (b) Percentage remaining supercoiled form *p < 0.05 when compared with control (t-test); **p < 0.05 when compared with H_2_O_2_ treatment alone (t-test)

### 3.5. Neuroprotective effects of O. asterosperma against glutamate toxicity in vitro

**Figure 4.**
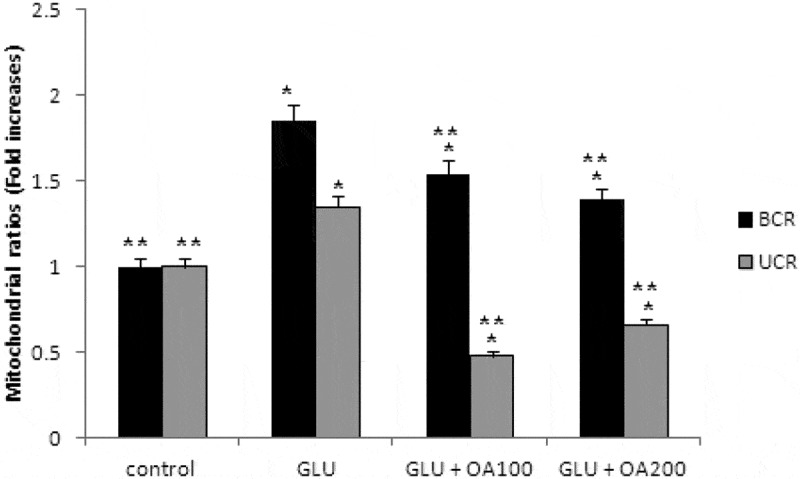
Effects of *Octaviania asterosperma extract* (100 and 200 µg/ml) and GLU on BCR and UCR ratios in HT22 cells

**Figure 5.**
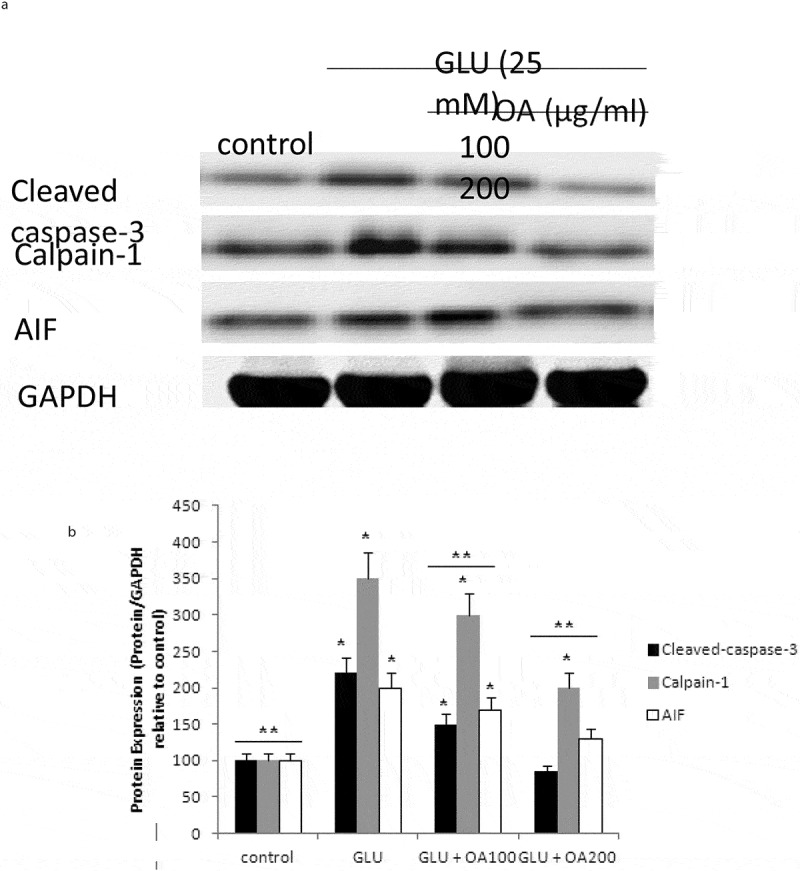
Apoptotic pathway in HT22 cell homogenates detected by Western blot analysis

**Figure 6.**
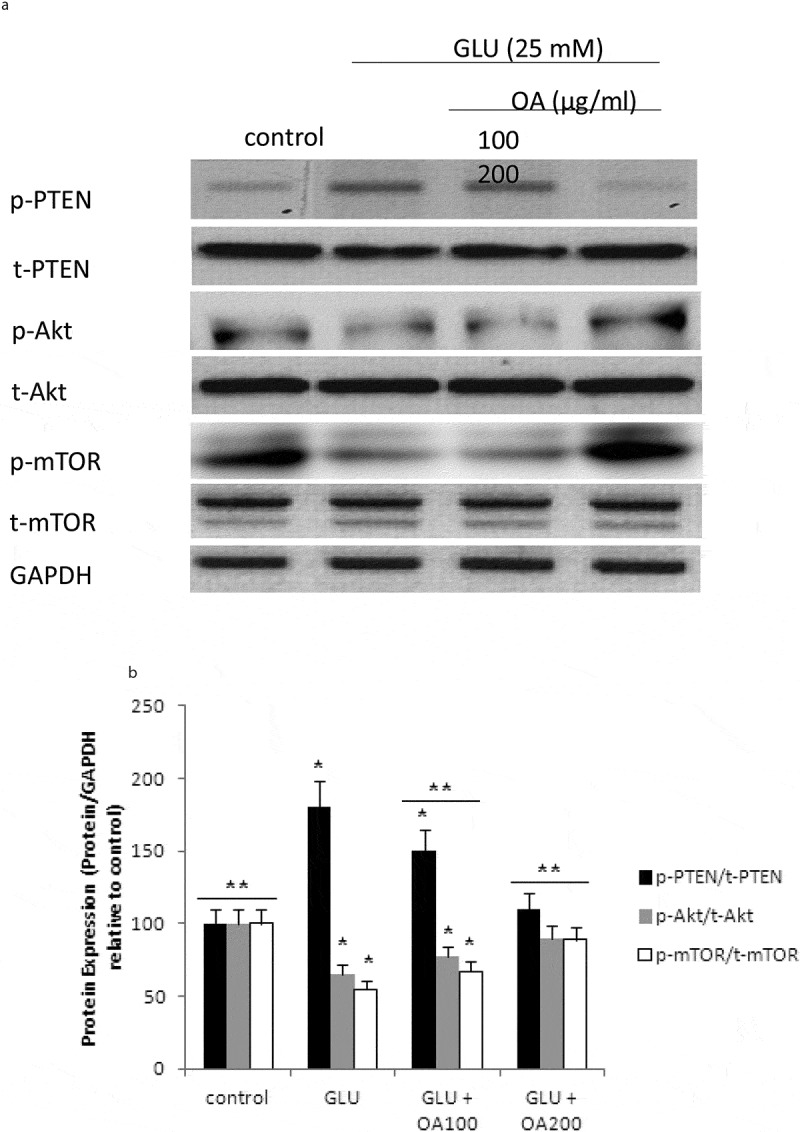
mTOR pathway in HT22 cell homogenates detected by Western blot analysis

Our study also investigated the effects of *O. asterosperma* against glutamate toxicity in HT22 cells. It is well established that excessive levels of GLU can inhibit cysteine uptake, reduce the levels of glutathione, and enhance oxidative stress and apoptosis (Chen et al. 2017; Lin et al. 2017; Park et al. 2017; Sadeghnia et al. 2017; Song et al. 2017; Tian et al. 2018). GLU can also lead to excessive activation of the NMDA receptor leading to increased Ca2+ influx (Trabelsi et al. 2017; Wang and Reddy 2017). Increased Ca2+-activated non-lysosomal cysteine proteases can lead to deregulation of calpain-1 activity and mitochondrial dysfunction (Cregan et al. 2004). The accumulation of ROS can further impair mitochondrial function (Tang et al. 2006). Our data shows that GLU can reduce the maximum mitochondrial respiration rate in HT22 cells, suggestive of mitochondrial malfunction due to impaired electron flow (Figure 4). We also show that methanol extracts could improve cell viability and improve the mitochondrial respiration rate, and regulate the expression of apoptotic protein (Figure 5)and the Akt/mTOR signalling pathway following exposure to GLU (Figure 6). More specifically, we report that *O. asterosperma* extracts inhibited the expression of cleaved caspase-3, which is activated in the cytoplasm due to GLU and can induce apoptosis. Activation of the Akt pathway in response to increased oxidative stress has been reported to enhance the phosphorylation of downstream mTOR and regulates cellular metabolism (Afanador et al. 2014; Kitagishi et al. 2014; Shaerzadeh et al. 2014; Cui et al. 2017; Matsuda et al. 2018). We report that *O. asterosperma* extract can inhibit the phosphorylation activity of PTEN. Another study recently showed that *Amanita caesarea*, an edible mushroom from Asia could protect against GLU-induced oxidative stress in HT22 cells via interaction with the Akt/mTOR pathway. The study also showed significant increases in rotarod endurance and decreased the escape latency time in the Morris water maze (a measure of memory) in an experimental AD mouse model. These changes were accompanied by increased in acetylcholine and choline acetyltransferase concentrations, and reduced ROS levels (Li et al. 2017). Taken together, it is likely that the neuroprotective effects of some mushrooms including *O. asterosperma* may also be due to modulation of the Akt/mTOR signaling pathways.

*p < 0.05 when compared with control (t-test); **p < 0.05 when compared with H_2_O_2_ treatment alone (t-test).

The levels of cleaved caspase-3, calpain-1, and AIF in neuronal cells exposed to *Octaviania asterosperma* and GLU. (a) The blot shown is representative tracings of an experiment done in triplicate. (b) Graphs are mean ± S.E for each treatment group. Each bar of the quantification graph represents the corresponding band for each treatment. Significance *p < 0.05 compared to control non-treated cells, **p < 0.05 compared to GLU-treated cells.

The levels of p-TEN, t-TEN, p-Akt, t-Akt, p-mTOR and t-mTOR in neuronal cells exposed to *Octaviania asterosperma* and GLU. (a) The blot shown is representative tracings of an experiment done in triplicate. (b) Graphs are mean ± S.E for each treatment group. Each bar of the quantification graph represents the corresponding band for each treatment. Significance *p < 0.05 compared to control non-treated cells, **p < 0.05 compared to GLU-treated cells.

## 4. Conclusion

This study is the first to report the biological activity in *O. asterosperma* mushroom collected in Bursa province (north-western Turkey). Our data suggests that this mushroom specie may be a vital food source rich in the determined phenolic, antioxidant and anti-inflammatory compounds. Our data also shows that this mushroom specie exhibited strong antimicrobial activity against other microorganisms. The extract lacks genotoxic potential and has excellent protection against GLU-induced toxicity *in vitro*. Further *in vivo* studies are required to valid the potential therapeutic role of *O. asterosperma* mushroom extracts in the clinic.
